# Multi‐omics of the expression and clinical outcomes of *TMPRSS2* in human various cancers: A potential therapeutic target for COVID‐19

**DOI:** 10.1111/jcmm.17090

**Published:** 2021-12-24

**Authors:** Li Liu, Ju‐Fang Qin, Man‐Zhen Zuo, Quan Zhou

**Affiliations:** ^1^ Department of Pediatrics The First College of Clinical Medical Science China Three Gorges University and Yichang Central People's Hospital Yichang China; ^2^ Department of Gynecology and Obstetrics The People's Hospital China Three Gorges University/the First People's Hospital of Yichang Yichang China

**Keywords:** bioinformatics, cancer progression, multi‐omics analysis, prognostic analysis, prognostic biomarkers, *TMPRSS2*

## Abstract

Growing evidence has shown that Transmembrane Serine Protease 2 (*TMPRSS2*) not only contributes to the severe acute respiratory syndrome coronavirus 2 (SARS‐CoV‐2) infection, but is also closely associated with the incidence and progression of tumours. However, the correlation of coronavirus disease (COVID‐19) and cancers, and the prognostic value and molecular function of *TMPRSS2* in various cancers have not been fully understood. In this study, the expression, genetic variations, correlated genes, immune infiltration and prognostic value of *TMPRSS2* were analysed in many cancers using different bioinformatics platforms. The observed findings revealed that the expression of *TMPRSS2* was considerably decreased in many tumour tissues. In the prognostic analysis, the expression of *TMPRSS2* was considerably linked with the clinical consequences of the brain, blood, colorectal, breast, ovarian, lung and soft tissue cancer. In protein network analysis, we determined 27 proteins as protein partners of *TMPRSS2*, which can regulate the progression and prognosis of cancer mediated by *TMPRSS2*. Besides, a high level of *TMPRSS2* was linked with immune cell infiltration in various cancers. Furthermore, according to the pathway analysis of differently expressed genes (DEGs) with *TMPRSS2* in lung, breast, ovarian and colorectal cancer, 160 DEGs genes were found and were significantly enriched in respiratory system infection and tumour progression pathways. In conclusion, the findings of this study demonstrate that *TMPRSS2* may be an effective biomarker and therapeutic target in various cancers in humans, and may also provide new directions for specific tumour patients to prevent SARS‐CoV‐2 infection during the COVID‐19 outbreak.

## 1 INTRODUCTION

Cancer is considered the most serious and prevalent disease in human beings around the world. The morbidity and mortality rate of cancer is the highest in the world. The 2020 cancer report estimates 19.3 million new cancer cases and 10.0 million cancer‐associated deaths.^1^ Owing to the increasing and ageing world population, the global cancer burden is expected to be 22.2 and 28.4 million new cases in 2030 and 2040, respectively, as depicted by the current trends.^1^, ^2^ In recent years, great efforts have been made for cancer prevention, screening, early detection, standardized treatment and regular follow‐up, however, the world still bears a large tumour burden due to the unclear pathogenesis of most tumours and the unavailability of potential biomarkers.^3^ It is crucial to extensively explore the pathogenesis of tumours and effective screening markers can be exploited as therapeutic targets.

Since the first case of severe acute respiratory syndrome coronavirus 2 (SARS‐CoV‐2) infections occurred in December 2019, the number of global coronavirus disease (COVID‐19) cases is still steadily increasing. As of 1 November 2021, the global COVID‐19 pandemic has led to 246,929,884 confirmed cases and 5,003,404 deaths over 188 countries/regions across the world.^4^ Clear evidence exists that patients with co‐morbidities are more susceptible to the COVID‐19, and are more likely to manifest complications and mortality after infection.^5^, ^6^ During the epidemic period, due to ageing, decreased immunity, delay in diagnosis, treatment and follow‐up, surgery, radiotherapy and chemotherapy and tumour‐related multiple co‐morbidities, cancer has been identified as an individual risk factor for COVID‐19.^6^, ^7^, ^8^, ^9^ A cohort study from China showed that the overall prevalence of COVID‐19 in cancer patients was considerably elevated than the overall incidence in the general population (1% vs. 0.29%).^10^, ^11^ In addition, various studies have reported that cancer patients are more likely to have serious clinical outcomes after suffering from COVID‐19.^7^, ^10^, ^12^, ^13^, ^14^ This might provide a clue that pays attention to the internal relationship between tumour and COVID‐19, which can lead to the preventing as well as controlling of COVID‐19 in cancer patients.

Transmembrane Serine Protease 2 (*TMPRSS2*) is a multifunctional encoding gene and is considered one of the members of the serine protease family. *TMPRSS2* contains four domains, that is, protease domain, type II transmembrane domain, receptor class A domain and scavenger receptor cysteine‐rich domain.^15^ Among them, the serine protease domain of the underlined protease cleaves, followed by secreting into the cell culture medium after being autocleavage. Thus, it participates in viruses in host cell processes.^15^, ^16^ *TMPRSS2* has been reported for its contribution to the process of human influenza viruses, coronaviruses including SARS‐CoV, SARS‐CoV‐2, Middle East respiratory syndrome coronavirus (MERS‐CoV) and human coronavirus 229E (HCoV‐229E) and entering host cells.^17^, ^18^ Currently, modulating the expression or activity of *TMPRSS2* is considered a potential intervention against human influenza viruses and coronaviruses including COVID‐19.^18^ On the other hand, multiple studies revealed that the expression of *TMPRSS2* was found to be considerably down‐regulated in tumour tissues compared to non‐tumorous ones, and abnormal expression of *TMPRSS2* was closely related to tumour growth, invasion, metastasis and prognosis in various cancers, especially prostate cancer.^19^, ^20^ More importantly, the Inhibition of *TMPRSS2* expression can reduce prostate or head and neck cancer cell invasion and metastasis, and reduce human lung Calu‐3 cells infection with SARS‐CoV‐2.^21^, ^22^ In addition, the *TMPRSS2* knockout mouse model in the cancer study showed that *TMPRSS2* inhibition is safe and effective for molecular therapy of tumours with few on‐target side effects.^23^ Therefore, a systematic and in‐depth investigation of the function of *TMPRSS2* in multiple tumours and COVID‐19 could pave the way for precision medicine and *TMPRSS2*‐targeted strategies.

Herein, to investigate the potential relationship between tumours and COVID‐19, and assess the expression level of *TMPRSS2* and its prognostic value in different carcinomas, we systematically studied the expression of *TMPRSS2* and its medical consequences in different types of carcinomas while employing multiple recognized online network databases. Furthermore, we examined the co‐altered genes with *TMPRSS2* for common cancer types and performed functional enrichment analysis. Therefore, the analyses may provide the potential value of *TMPRSS2* expression for the survival of patients associated with cancer, and give potential direction to prevent COVID‐19 pandemic for specific tumour patients.

## 2 MATERIALS AND METHODS

### 2.1 Analyses of Oncomine dataset

In this study, a public web‐based microarray database Oncomine (www.oncomine.org) was employed for the analysis of the *TMPRSS2* transcription levels in cancerous specimens followed by comparing the obtained results with the healthy specimens (controls).^24^ The thresholds were restricted in the following manner: fold‐change = 1.5; *p* = 0.001; data type: mRNA.

### 2.2 Analysis of GEPIA dataset

Gene Expression Profiling Interactive Analysis (GEPIA) (http://gepia.cancer‐pku.cn) offers significant interactive and customizable tasks, such as profile plotting, differential expression analysis, correlation analysis, estimating RNA sequencing expression data based on 8587 healthy and 9736 cancer samples in Genotype–Tissue Expression (GTEx) and TCGA projects.^25^ We used GEPIA to verify the differences in *TMPRSS2* gene expression in both healthy and different types of cancer tissues. In addition, profile plotting based on cancer pathological stage or type of cancer, survival rate of patient, similar gene detection, correlation analyses and dimensionality reduction analysis can be carried out via the GEPIA dataset.

### 2.3 UALCAN database analysis

UALCAN (http: //ualcan. path.uab.edu/index.html) is a user‐friendly integrated data‐mining platform and is used for the extensive analysis of data obtained from cancer OMICS.^26^ It is built on PERL‐CGI and can also be employed for gene expression analysis, methylation of promoter, prognosis and correlation. In this study, the UALCAN database was employed for analysing the expression pattern and promoter methylation profiling of *TMPRSS2* mRNA.

### 2.4 cBioPortal dataset and muTarget database analysis

TCGA (cancergenome.nih.gov/) is a comprehensive database, which has both sequencing and pathological data of 30 various forms of carcinomas. For cancer genomics, cBioPortal (http://www.cbioportal.org/) is an open‐access platform, which can be utilized for the multi‐functional visualization of complex cancer genomics, integrative analysis and clinical profiling.^27^ We employed cBioPortal to evaluate the *TMPRSS2* alterations frequency, mRNA expression *z*‐scores (RNA Seq V2 RSEM) and copy number variance via subtypes of each carcinoma from the TCGA PanCanAtlas dataset. To further clarify the relationship between *TMPRSS2* gene mutation and protein expression, we used the muTarget database (https://www.mutarget.com/) to analyse the effect of *TMPRSS2* gene mutation on protein expression levels in different tumour types. The cut‐off *p*‐value was regarded as <0.01.^28^

### 2.5 TIMER analysis

TIMER (https://cistrome.shinyapps.io/timer/) database was employed to validate the *TMPRSS2* expression level in different form of carcinomas,^29^ followed by estimating the Spearman's correlation analysis between the *TMPRSS2* expression levels and six immune infiltrates, such as CD8+ T cells, CD4+ T cells, B cells, macrophages, dendritic cells (DCs) and neutrophils obtained from four common carcinomas. The expression scatter plots were formed between a pair of user‐defined genes in a given cancer type by using a correlation module, followed by revealing the level of gene expression via log2 RSEM.

### 2.6 PrognoScan database analysis

An online database PrognoScan (http://dna00.bio.kyutech.ac.jp/PrognoScan/) provides a platform to assess effective tumour biomarkers and their significant therapeutic targets.^30^ PrognoScan was employed for exploring the correlation between the *TMPRSS2* expression and survival rate of patients in different carcinomas. According to the obtained results, the threshold was found to be Cox *p* < 0.05.

### 2.7 The Kaplan–Meier plotter analysis

A web‐based online database Kaplan–Meier plotter (www.kmplot.com) is used to study prognostic implications of genes in various forms of carcinoma. The underlined database comprised data, associated with the rate of survival and gene expression in 7461 samples, obtained from 21 different types of tumour.^31^ We employed this database for the validation of the *TMPRSS2* prognostic value in different types of cancers, with an HR with 95% CI and log‐rank *p*‐value.

### 2.8 Protein–Protein interaction analysis

An online interface GeneMANIA (https://genemania.org/) is a user‐friendly data mining platform. It can be used for the generation of genes correlated to a set of input genes, based on protein and genetic interactions, pathways, co‐localization, co‐expression and protein domain similarity.^32^ STRING (https://string‐db.org/) is associated with protein–protein interactions.^33^ Herein, we employed both the GeneMANIA and STRING servers to explore the related genes of *TMPRSS2*.

### 2.9 Co‐expressed and pathway analysis

The R2: Genomics Analysis and Visualization Platform‐V‐3.2.0 (https://hgserver1.amc.nl/) was employed for the integrative analysis of the positively and negatively co‐expressed genes of *TMPRSS2* in TCGA dataset of four different forms of carcinomas, that is, colorectal, breast, lung and ovarian, and the cut‐off *p*‐value was regarded as <0.01. The co‐expressed genes in the four common tumours are obtained through the intersection of the Venn diagram. Then, we used the React me tool (https://reactome.org/) to explore pathways shared by TMPRSS2‐correlated genes and subsequently categorized them according to their KEGG pathway.^34^

## 3 RESULTS

### 3.1 The expression level of *TMPRSS2*in many cancers

**FIGURE 1:**
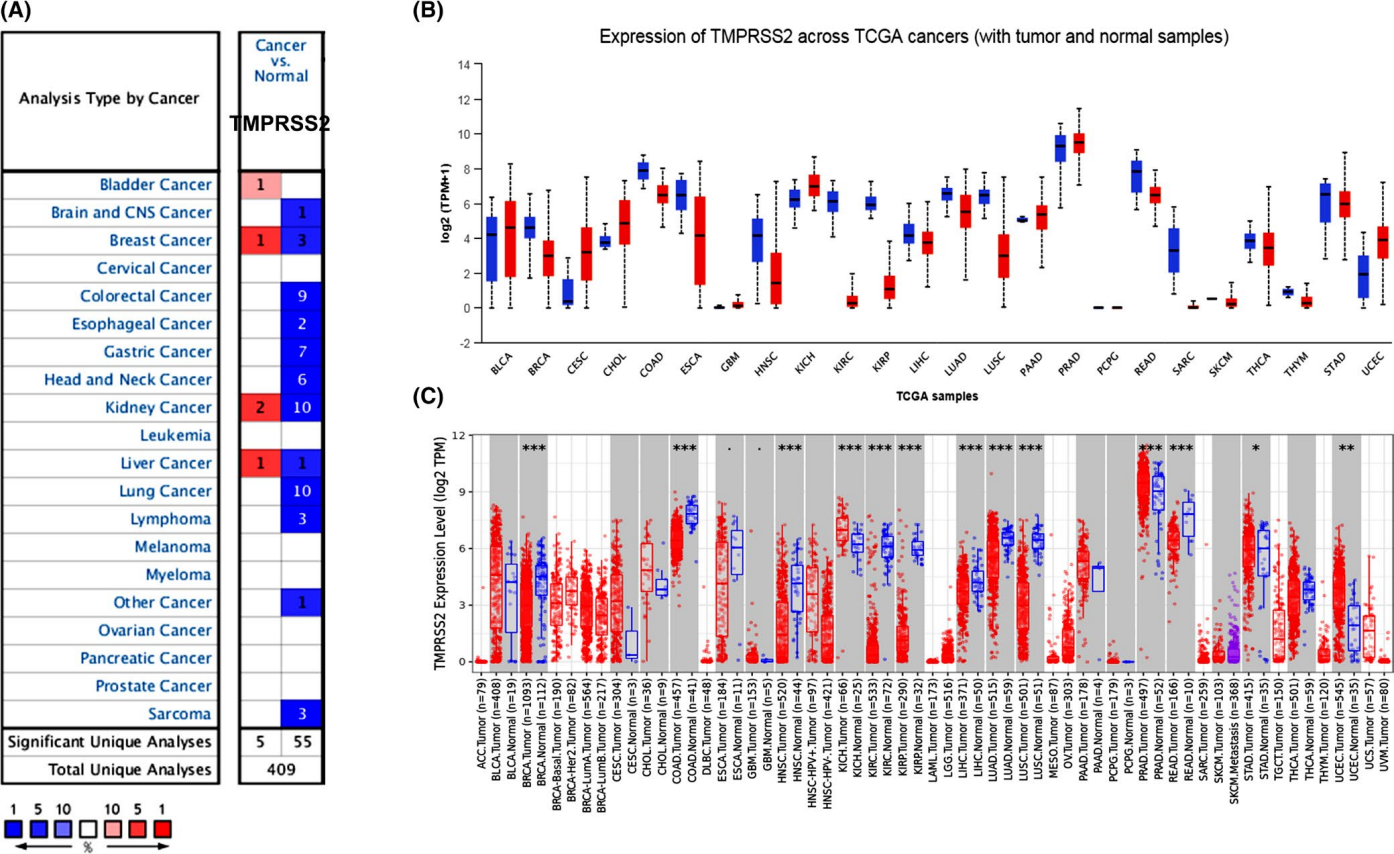
Expression pattern of *TMPRSS2* mRNA in many cancers by Oncomine, UALCAN and GEPIA databases. (A) The expression pattern of *TMPRSS2* mRNA in various cancers was searched in the Oncomine database. The underlined graphic was generated from the Oncomine database, revealing the number of datasets (*p* < 0.01) mRNA over (red) or under expression (blue) of *TMPRSS2* (tumour tissue vs. corresponding normal tissue). The threshold was considered with the underlined parameters: *p* and fold‐change were equal to 0.001 and 1.5, respectively, and the data type was mRNA. (B) The expression of *TMPRSS2* mRNA in many cancers was searched in the UALCAN database. Boxes represent the median and the 25th and 75th percentiles; green and red boxes indicate normal and tumour tissues respectively. (C) The expression level of *TMPRSS2* mRNA in many cancers was searched in the TIMER database. Boxes indicate the median and the 25th and 75th percentiles; blue and red boxes indicate normal and tumour tissues respectively. Blue and red dashed lines indicate the average value of normal and tumour tissues respectively. *p = 0.05，**p = 0.01, ***p = 0.001

Databases including Oncomine and TCGA were employed for the evaluation of *TMPRSS2* differential expression patterns in many kinds of cancer by UALCAN and GEPIA. The results obtained from the Oncomine database indicated that relative to normal tissues, the expression level of *TMPRSS2* was reduced in many tumour tissues and cancers including breast, bladder, gastric, colorectal, lung, kidney, prostate, ovarian and sarcoma cancer. There is only one dataset study that shows an elevated expression of *TMPRSS2* in breast, kidney and liver cancer, respectively, as depicted in Figure 1A. The UALCAN, TIMER and GEPIA were used to further explore the *TMPRSS2* expression in various cancers. The results of the UALCAN and TIMER databases indicated that the expression of *TMPRSS2* was decreased in many kinds of cancer, that is, colon adenocarcinoma (COAD), head and neck squamous cell carcinoma (HNSC), breast invasive carcinoma (BRCA), kidney renal papillary cell carcinoma (KIRP), oesophageal carcinoma (ESCA), lung adenocarcinoma (LUAD), kidney renal clear cell carcinoma (KIRC), rectum adenocarcinoma (READ), liver hepatocellular carcinoma (LIHC), sarcoma (SAEC), lung squamous cell carcinoma (LUSC), thyroid carcinoma (THCA), skin cutaneous melanoma (SKCM), stomach adenocarcinoma (STAD) and thymoma (THYM). However, elevated expression of *TMPRSS2* was reported in bladder urothelial carcinoma (BLCA), cervical squamous cell carcinoma and endocervical adenocarcinoma (CESC), cholangiocarcinoma (CHOL), glioblastoma multiforme (GBM), kidney chromophobe (KICH), pancreatic adenocarcinoma (PAAD), prostate adenocarcinoma (PRAD) and uterine corpus endometrial carcinoma (UCEC; Figure 1B,C, Figure S1). The results of the GEPIA database are similar to the results of Oncomine, suggesting that the expression of *TMPRSS2* was considerably reduced in COAD, KICH, BRCA, HNSC, KIRP, LUAD, KIRC, LUSC and LIHC tumour tissues, however, *TMPRSS2* expression was considerably elevated in the UCEC, as depicted in Figure S2.

### 3.2 *TMPRSS2* promoter methylation in many cancers

**FIGURE 2:**
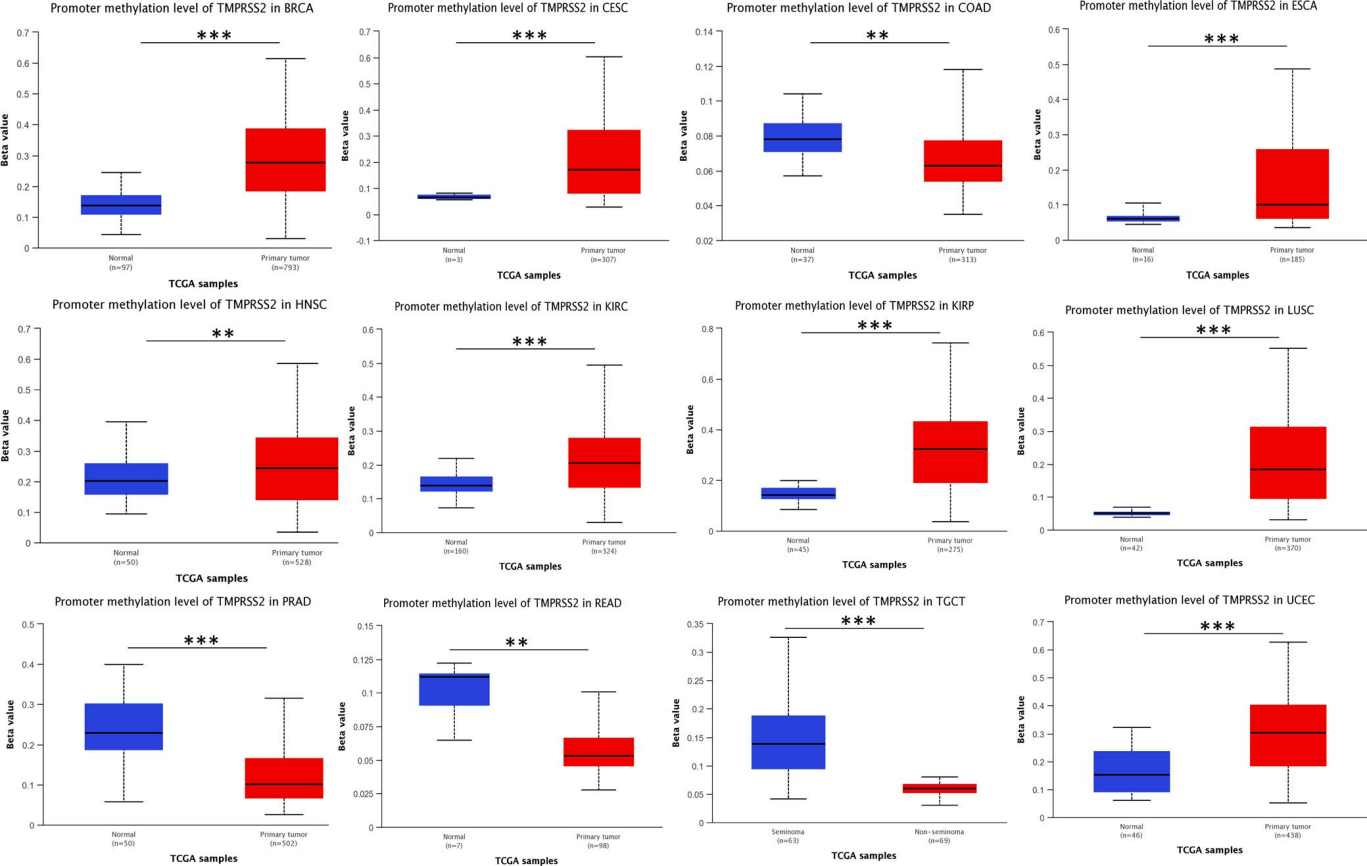
*TMPRSS2* promoter methylation in various cancers using the UALCAN database. The methylation levels of *TMPRSS2* promoter in BRCA, CESC, ESCA, HNSC, KIRC, KIRP and UCEC were considerably elevated than that in normal tissue. On the contrary, the methylation levels of *TMPRSS2* promoter in COAD, PRAD, READ and TGCT were slightly reduced relative to that in normal tissue. Boxes indicate the median and the 25th and 75th percentiles; green and red boxes show normal and tumour tissues respectively. Green and red dashed lines indicate the average value of all normal and tumour tissues respectively. **p = 0.001, ***p = 0.001

Due to the lower expression of *TMPRSS2* in a variety of tumour tissues, we evaluated the gene promoter of *TMPRSS2*. UALCAN database was used to verify the level of methylation of *TMPRSS2* promoter in various cancers. The results of the UALCAN database showed that the methylation levels of *TMPRSS2* promoter in BRCA, CESC, ESCA, HNSC, KIRC, KIRP and UCEC were considerably elevated relative to that in normal tissue, as depicted in Figure 2. In contrast, the methylation levels of *TMPRSS2* promoter in COAD, PRAD, READ and testicular germ cell tumours (TGCT) were relatively decreased than those in normal tissue. *TMPRSS2* promoter methylation level was negatively correlated with gene expression level, it is indicated that *TMPRSS2* promoter hypermethylation in various cancers may trigger itself and elevates its level accordingly.

### 3.3 *TMPRSS2* genetic variation in various cancers

**FIGURE 3:**
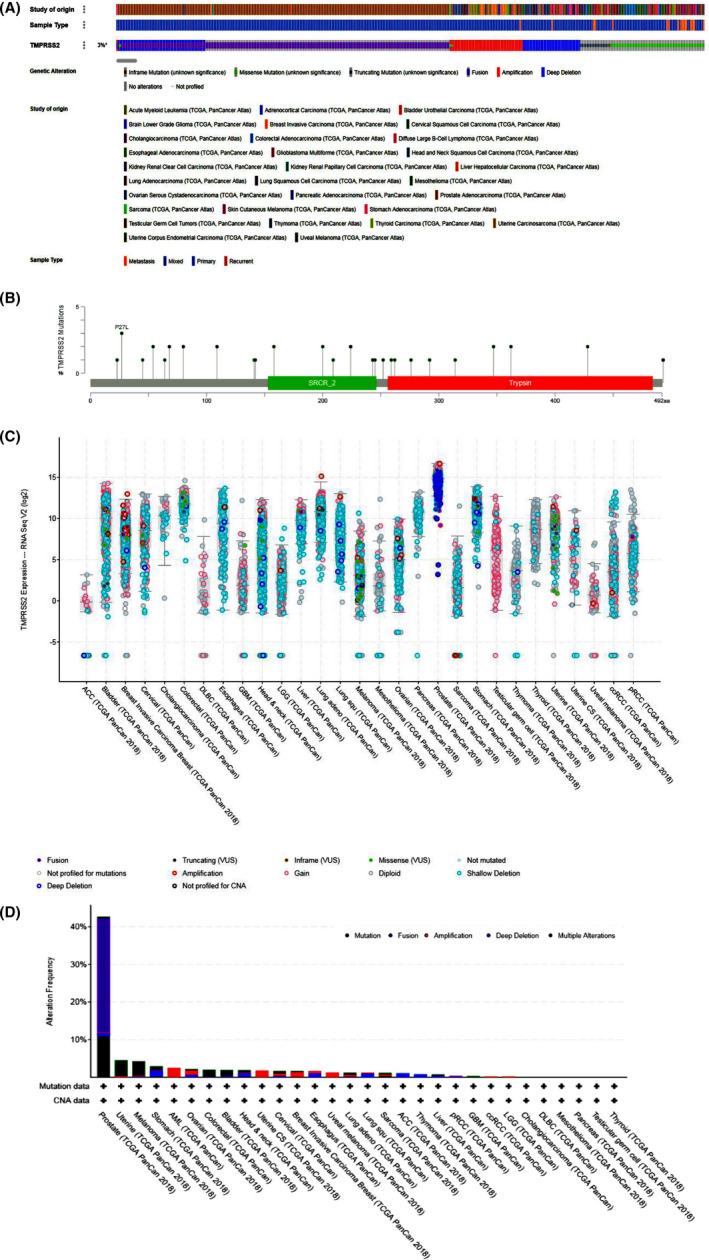
Frequency of mutations, CNAs and expression in many cancers obtained from cBioPortal web. (A) *TMPRSS2* was only altered in 371 (3%) of 10,953 queried patients in 371 (3%) of 10,967 queried samples. (B) Total 329 mutations were found within amino acids 1 to 492 of *TMPRSS2*. Among them, there are 7 missense mutation sites, 19 truncating sites, 11 inframe mutation sites and 242 fusion mutation sites respectively. Mutation sites were found in a hotspot in SRCR_2 and trypsin domains. (C) In 29 cancer studies, the expression of *TMPRSS2* mRNA (RNA Seq V2) was generated from the cBioPortal web. The *x*‐axis has been categorized based on cancer type and *y*‐axis indicates the expression level of BMP5 mRNA. The expression frequency revealed fusions (violet), missense mutations (green), no mutations (blue) and truncating (deep blue). (D) Among the 32 datasets, the percentage of *TMPRSS2* alteration frequency varied from 0% to 42.7% in many cancers. The highest alteration frequency was found in prostate cancer (42.7%), whereas other types of tumours all exhibited very low mutation alteration (<10%) among all of the query cancer samples. The alteration frequency revealed fusions (violet), mutations (green) and multiple

**FIGURE 4:**
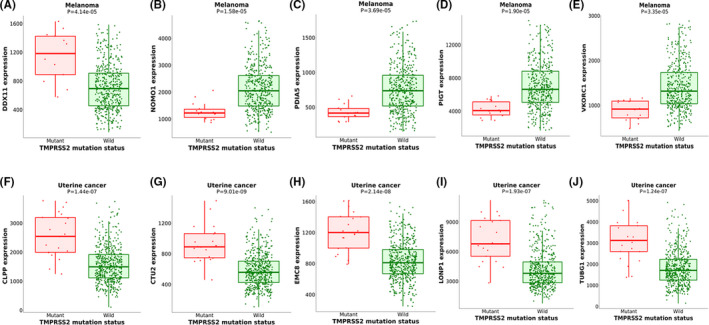
The effect of *TMPRSS2* gene mutation on the expression of the five most significant genes in melanoma and uterine cancer obtained from muTarget database. Histogram of the effect of *TMPRSS2* gene mutation on the expression of (A) *DDX11* in melanoma, (B) *NOMO1* in melanoma, (C) *PDIA5* in melanoma, (D) *PIGT* in melanoma, (E) *VKORC1* in melanoma, (F) *CLPP* in uterine cancer, (G) *CTU2* in uterine cancer, (H) *EMC8* in uterine cancer, (I) *LONP1* in uterine cancer and (J) *TUBG1* in uterine cancer. Boxes indicate the median and the 25th and 75th percentiles. Green and red boxes indicate wild and mutant tissues. Green and red dashed lines indicate the average value of wild and mutant tissues respectively

The gene mutations of *TMPRSS2* were explored in 32 common types of the tumour by the TCGA PanCancer Atlas database, mainly including mutation and copy number mutation. As showed in Figure 3, *TMPRSS2* was only altered in 371 (3%) of 10,953 queried patients and 371 (3%) of 10,967 queried samples. Total 329 mutations were present within amino acids 1 to 492aa of *TMPRSS2*. Among them, there are 7 missense mutation sites, 19 truncating sites, 11 inframe mutation sites and 242 fusion mutation sites respectively. Mutation sites were located in a hotspot in SRCR_2 and trypsin domains. Among the 32 datasets, the percentage of *TMPRSS2* alteration frequency varied from 0% to 42.7% in various cancers. The highest alteration frequency was found in prostate cancer (42.7%), whereas other types of tumours exhibited very low mutation alteration (<10%) among all of the query cancer samples. In addition, our research found that the gene mutation of *TMPRSS2* cannot cause changes in its own expression, but it can lead to the differential expression of multiple genes in melanoma and uterine cancer. The most significant differences in melanoma are *NOMO1*, *PIGT*, *VKORC1*, *PDIA5* and *DDX11*, and the most significant differences in uterine cancer are *CTU2*, *EMC8*, *TUBG1*, *CLPP* and *LONP1*. The details are shown in Figure 4. The mutation frequency of the *TMPRSS2* gene is not high in most tumour types, suggesting that the mutation of the *TMPRSS2* gene itself may have little effect on the *TMPRSS2* gene function.

### 3.4 *TMPRSS2* gene and protein partners in various cancers

**FIGURE 5:**
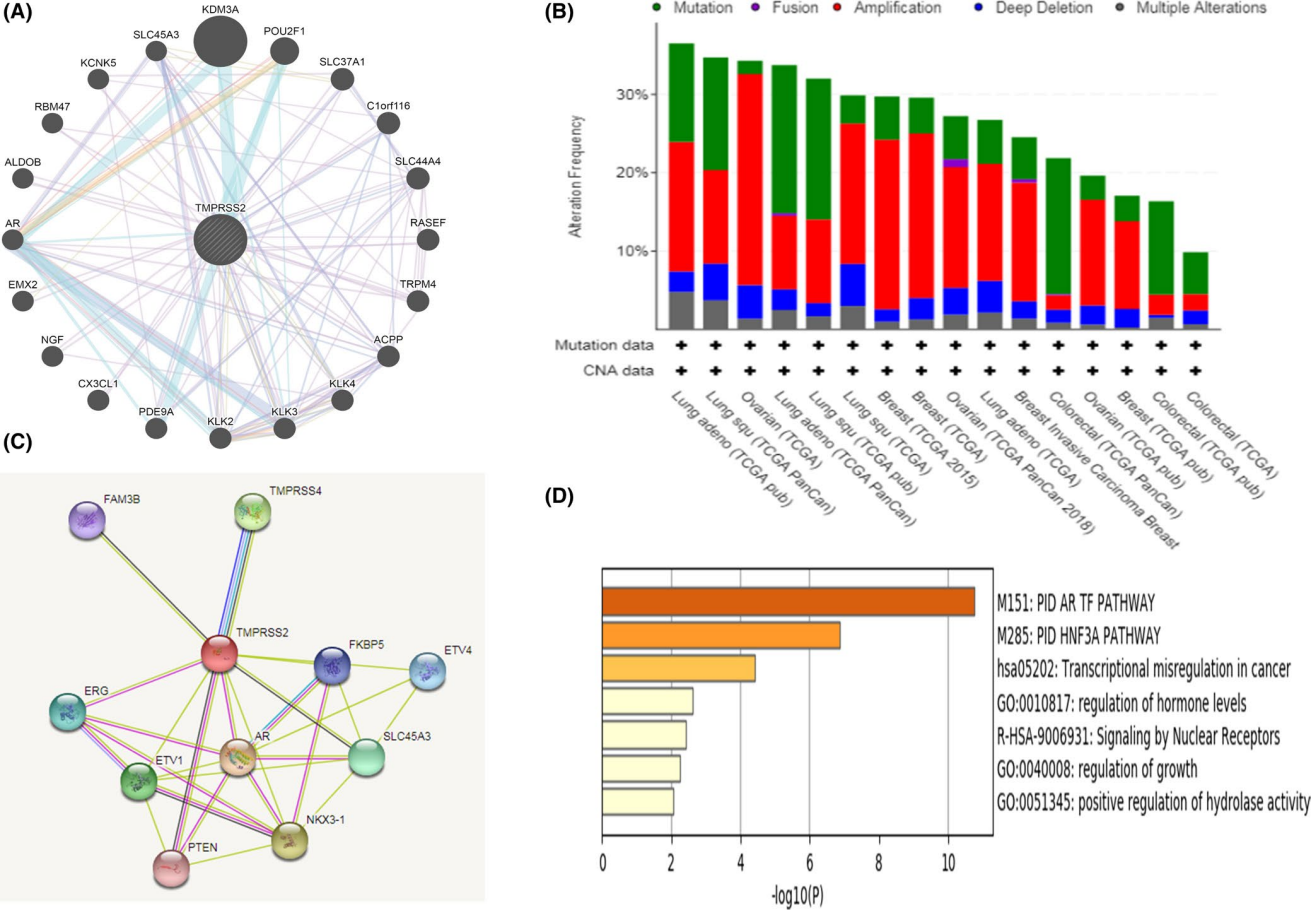
Interaction and co‐occurrence functional protein partners of *TMPRSS2*. (A) Expected functional partners of *TMPRSS2* generated from GeneMANIA. Circles displayed depicting nodes. Predicted functional partners are indicated post observing co‐localization, co‐expression, genetic and physical interactions, cascades and shared protein domains (predicted). (B) Predicted functional partners of *TMPRSS2* generated from STRING. Circles revealed indicating nodes. Predicted functional partners are indicating post considering physical and functional associations. (C) The alteration frequency of 27 partners gene signature using cBioPortal web. The highest alteration frequency was observed in lung squamous carcinoma (>30%). Green depicts the mutations in alteration frequency, while violet, red, deep blue and grey colours depict fusions, amplifications, deep deletions and multiple alterations respectively. (D) The enrichment analysis of 27 partners gene signature. The obtained results revealed that the underlined genes are largely enriched in PID AR TF and PID HNF3A pathways, transcriptional misregulation in cancer, regulation of hormone levels, signalling by nuclear receptors, regulation of growth and positive regulation of hydrolase activity (*p* < 0.05)

GeneMANIA web was used for the prediction of the functionally similar genes including *TMPRSS2*, which accumulates data on co‐localization, co‐expression, genetic interactions, involved cascades, prediction of physical interactions and shared protein domains, as depicted in Figure 5A. The predicted functionally similar genes of *TMPRSS2* were *KDM3A*, *POU2F1*, *SLC37A1*, *C1orf116*, *SLC44A4*, *RASEF*, *TRPM4*, *ACPP*, *KLK4*, *KLK3*, *KLK2*, *PDE9A*, *CX3CL1*, *NGF*, *EMX2*, *AR*, *ALDOB*, *RBM47*, *KCNK5* and *SLC45A3*. At the same time, the protein–protein interaction network of the *TMPRSS2* was predicted by the STRING database (Figure 5C). It was found that 11 proteins interacted with *TMPRSS2* proteins. The predicted protein partners of *TMPRSS2* were *FKBP5*, *AR*, *NKX3*‐*1*, *TMPRSS4*, *ETV1*, *ERG*, *SLC45A3*, *PTEN*, *ETV4* and *FAM3B*. Hence, 27 predicted genes and proteins associated with *TMPRSS2* might have a role in regulating cancer development (mediated by *TMPRSS2*) and prognosis. The mutations and copy number alterations (CNAs) were analysed in the 27 predicted–associated genes of *TMPRSS2* via the cBioPortal database. There was an alteration in queried genes of 3225 (38%) out of 10,953 queried patients. The highest alteration frequency was observed in lung squamous carcinoma (>30%), while the lowest alteration (<10%) was observed in colorectal cancer among all of the query cancer samples, as depicted in Figure 5B. Enrichment analysis revealed that the underlined genes were commonly enriched in *PID AR TF* and *PID HNF3A* pathways, transcriptional misregulation in cancer, regulation of hormone levels, signalling by nuclear receptors, regulation of growth and positive regulation of hydrolase activity, as depicted in Figure 5D.

### 3.5 Prognostic analysis of *TMPRSS2* in many cancers

**FIGURE 6:**
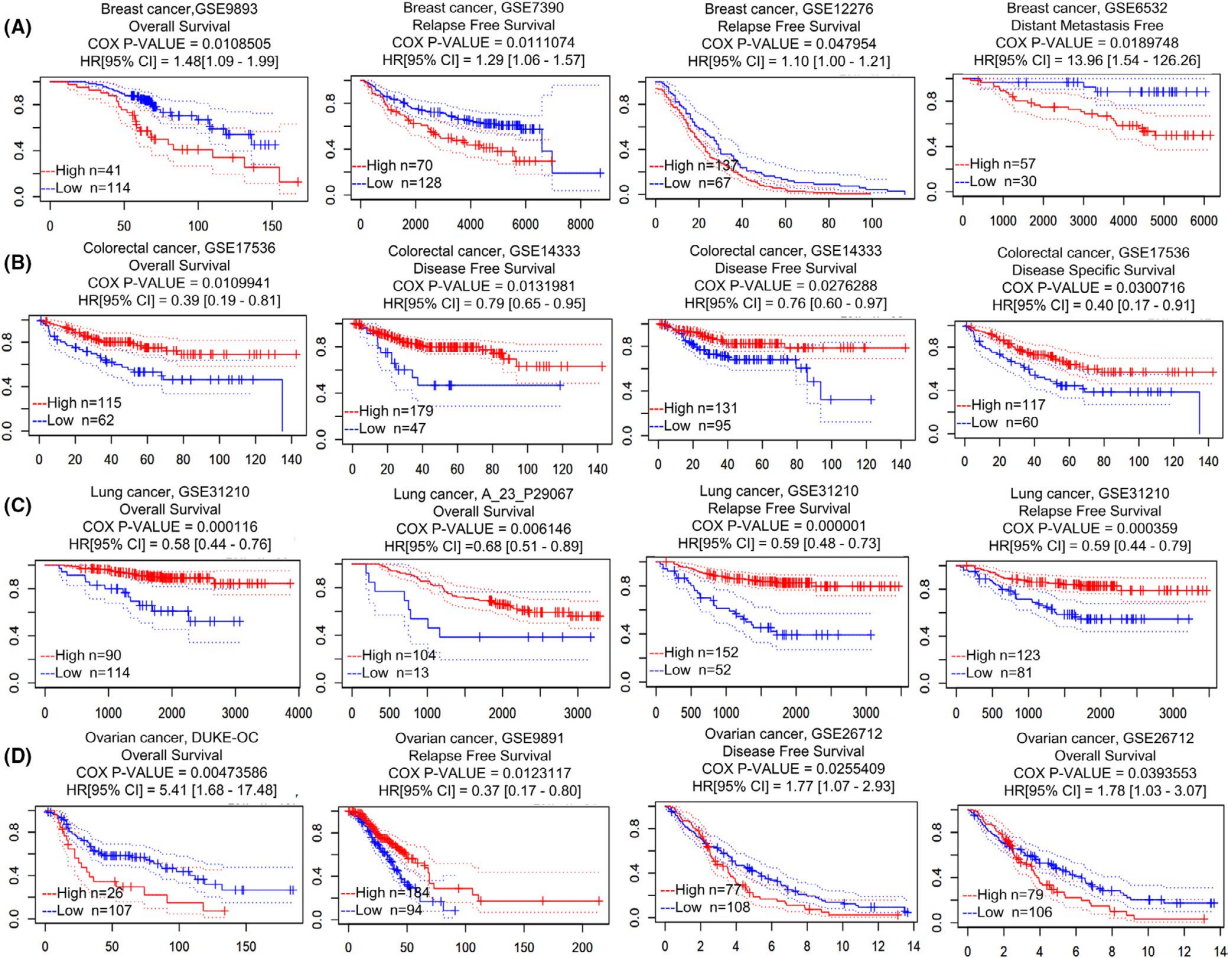
Association between the expression level of *TMPRSS2* and prognosis in four common kinds of cancers, that is, lung, breast, ovarian and colorectal cancer by PrognoScan database. The survival curve comparing patients with an elevated (red) and reduced (blue) expression in the breast (A), colorectal (B), lung (C) and ovarian cancer (D) that were retrieved from the PrognoScan database. Survival curve analysis was carried out by threshold Cox *p* < 0.05. The dotted lines indicate maximum and minimum values of the survival average

We explored the prognostic value of *TMPRSS2* mRNA expression in many types of cancers by PrognoScan and Kaplan–Meier plotter databases. The PrognoScan database results revealed that the *TMPRSS2* expression level was considerably linked with the prognosis of brain, blood, colorectal, breast, ovarian, lung and soft tissue cancer. In breast cancer, GSE9893, GSE7390, GSE12276 and GSE6532 datasets revealed that the patients with up‐regulated *TMPRSS2* expression had significantly poor overall survival (OS), relapse‐free survival (RFS) and distant metastasis‐free survival (DMFS) compared to patients with lower *TMPRSS2* expression (Figure 6A and Table 1). The opposing results were found in colorectal cancer datasets. The GSE17536, GSE14333 and GSE17536 datasets revealed that the patient's group with an elevated level of *TMPRSS2* mRNA expression had significantly better OS and disease‐free survival (DFS) than the low expression group (Figure 6B and Table 1). Analysis of GSE31210 and GSE13213 datasets of PrognoScan revealed considerably poor OS and RFS of lung cancer patients in the down‐regulated *TMPRSS2* mRNA expression group relative to their elevated expression counterparts (Figure 6C and Table 1). High OS and DFS ratio were shown in the low *TMPRSS2* expression group of ovarian cancer relative to the elevated expression group, according to the DUKE‐OC, GSE9891 and GSE26712 (205102_at) datasets, while one alteration revealed by dataset GSE26712 (211689_s_at) reversed the correlation of reduced expression with poor DFS of ovarian cancer patients, as depicted in Figure 6D and Table 1.

**TABLE 1:** Prognosis analysis of *TMPRSS2* expression in various cancer patients (PrognoScan database)

Dataset	Cancer type	Endpoint	Cohort	Contributor	Probe ID	*N*	Cox *p*	Ln (HR)	HR (95% CI)
GSE31210	Lung cancer	RFS	NCCRI	Okayama	1570433_at	204	8.80481E−07	−0.525474	0.59 (0.48–0.73)
GSE31210	Lung cancer	OS	NCCRI	Okayama	1570433_at	204	0.000115681	−0.551071	0.58 (0.44–0.76)
GSE31210	Lung cancer	RFS	NCCRI	Okayama	205102_at	204	0.000358629	−0.527135	0.59 (0.44–0.79)
DUKE‐OC	Ovarian cancer	OS	Duke	Bild	205102_at	133	0.00473586	1.68887	5.41 (1.68–17.48)
GSE31210	Lung cancer	OS	NCCRI	Okayama	205102_at	204	0.00581929	−0.520625	0.59 (0.41–0.86)
GSE13213	Lung cancer	OS	Nagoya	Tomida	A_23_P29067	117	0.00614575	−0.3926	0.68 (0.51–0.89)
GSE31210	Lung cancer	RFS	NCCRI	Okayama	226553_at	204	0.00700726	−0.267109	0.77 (0.63–0.93)
GSE9893	Breast cancer	OS	Montpellier, Bordeaux, Turin	Chanrion	13277	155	0.0108505	0.388917	1.48 (1.09–1.99)
GSE17536	Colorectal cancer	OS	MCC	Smith	205102_at	177	0.0109941	−0.945643	0.39 (0.19–0.81)
GSE7390	Breast cancer	RFS	Uppsala, Oxford, Stockholm, IGR, GUYT, CRH	Desmedt	205102_at	198	0.0111074	0.255479	1.29 (1.06–1.57)
GSE9891	Ovarian cancer	OS	AOCS, RBH, WH, NKI‐AVL	Tothill	205102_at	278	0.0123117	−1.00512	0.37 (0.17–0.80)
GSE14333	Colorectal cancer	DFS	Melbourne	Jorissen	226553_at	226	0.0131981	−0.240839	0.79 (0.65–0.95)
Jacob−00182‐UM	Lung cancer	OS	UM	Shedden	211689_s_at	178	0.0143181	−0.262144	0.77 (0.62–0.95)
GSE30929	Soft tissue cancer	DRFS	MSKCC (1993–2008)	Gobble	205102_at	140	0.0166028	−2.50239	0.08 (0.01–0.63)
GSE6532‐GPL570	Breast cancer	RFS	GUYT	Loi	1570433_at	87	0.0189748	2.63604	13.96 (1.54–126.26)
GSE6532‐GPL570	Breast cancer	DMFS	GUYT	Loi	1570433_at	87	0.0189748	2.63604	13.96 (1.54–126.26)
GSE26712	Ovarian cancer	DFS	MSKCC	Bonome	211689_s_at	185	0.0255409	0.573233	1.77 (1.07–2.93)
GSE4412‐GPL96	Brain cancer	OS	UCLA	Freije	211689_s_at	74	0.0271724	0.46614	1.59 (1.05–2.41)
GSE14333	Colorectal cancer	DFS	Melbourne	Jorissen	1570433_at	226	0.0276288	−0.274568	0.76 (0.60–0.97)
GSE17536	Colorectal cancer	DSS	MCC	Smith	205102_at	177	0.0300716	−0.926539	0.40 (0.17–0.91)
GSE2658	Blood cancer	DSS	Arkansas	Zhan	211689_s_at	559	0.0317345	−0.416225	0.66 (0.45–0.96)
GSE4412‐GPL97	Brain cancer	OS	UCLA	Freije	226553_at	74	0.0361025	1.22568	3.41 (1.08–10.72)
Jacob−00182‐MSK	Lung cancer	OS	MSK	Shedden	205102_at	104	0.0362315	−1.0361	0.35 (0.13–0.94)
GSE26712	Ovarian cancer	OS	MSKCC	Bonome	211689_s_at	185	0.0393553	0.574689	1.78 (1.03–3.07)
Jacob−00182‐HLM	Lung cancer	OS	HLM	Shedden	211689_s_at	79	0.0419985	−0.290393	0.75 (0.57–0.99)
GSE4573	Lung cancer	OS	Michigan	Raponi	211689_s_at	129	0.0421505	−0.181691	0.83 (0.70–0.99)
GSE12276	Breast cancer	RFS	EMC	Bos	211689_s_at	204	0.0479541	0.0962522	1.10 (1.00–1.21)

Abbreviations: 95% CI, confidence interval; DFS, disease‐free survival; DMFS, distant metastasis‐free survival; DRFS, distant recurrence‐free survival; DSS, disease‐specific survival; HR, hazard rate; OS, overall survival; RFS, relapse‐free survival.

**FIGURE 7:**
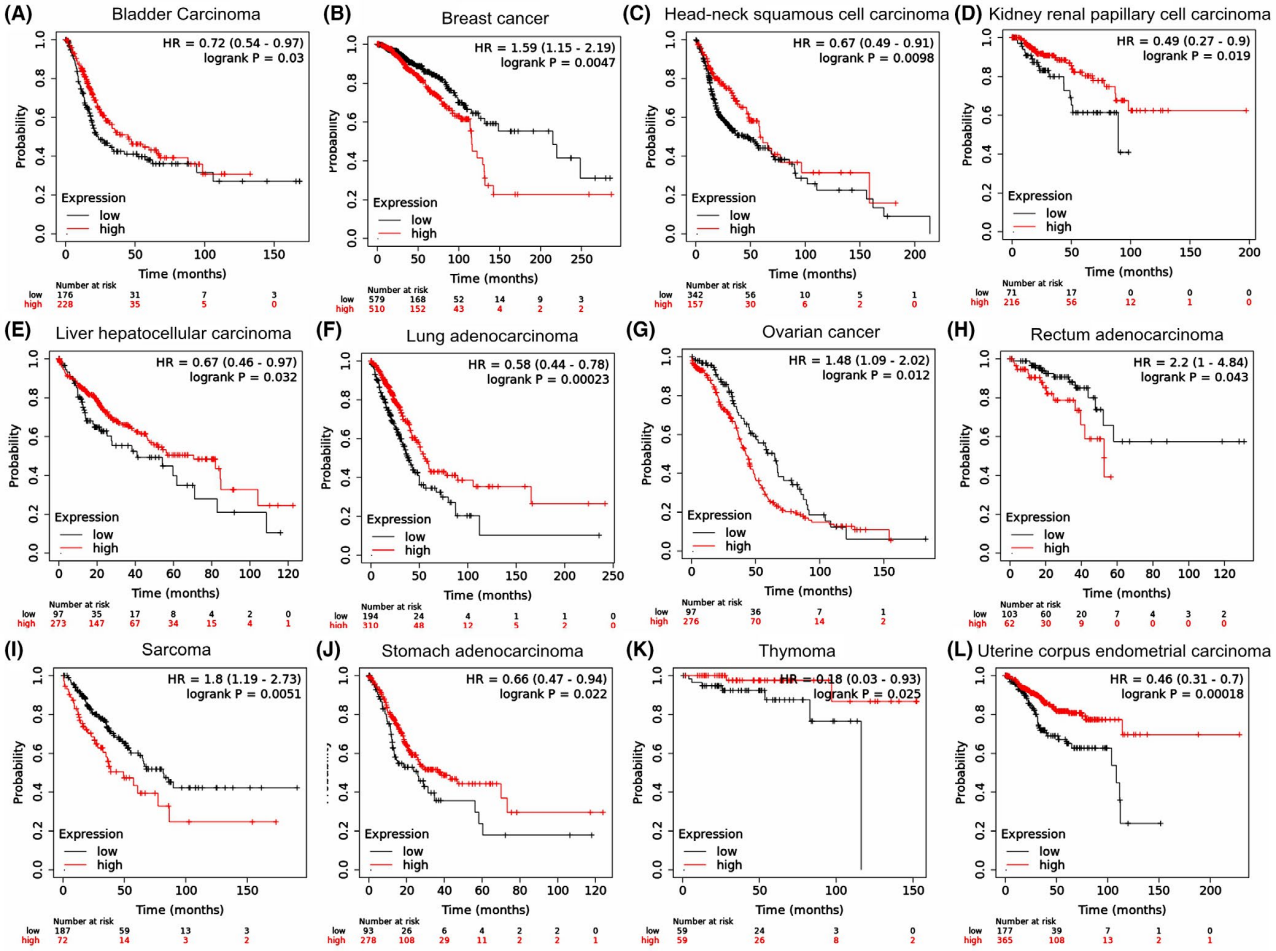
Confirmation of the association between the expression level of *TMPRSS2* and prognosis in many cancer patients by Kaplan–Meier plotter database. The survival curve comparing patients with an elevated (red) and reduced (blue) expression in bladder carcinoma (A), breast carcinoma (B), head–neck squamous cell carcinoma (C), kidney renal papillary cell carcinoma (D), liver hepatocellular carcinoma (E), lung adenocarcinoma (F), ovarian cancer (G), rectum adenocarcinoma (H), sarcoma (I), stomach adenocarcinoma (J), thymoma (K) and uterine corpus endometrial carcinoma (L) were retrieved from the Kaplan–Meier plotter database. Survival curve analysis was performed by threshold Cox *p* < 0.05. The dotted lines indicate maximum and minimum values of the survival average

Kaplan–Meier plotter database was employed to verify the prognosis of *TMPRSS2* in various types of cancer based on 7489 patient data. The obtained data indicated that an elevated expression level of *TMPRSS2* was linked with significant OS prognosis in patient with bladder, head–neck squamous cell, kidney renal papillary cell, liver hepatocellular, lung adeno, stomach adeno and uterine corpus endometrial carcinoma as well as thymoma, whereas the association of higher expression with bad DFS prognosis in patient with breast cancer, ovarian cancer, rectum adenocarcinoma and sarcoma (Figures 6 and 7 and Table 2). In addition, the Kaplan–Meier plot indicated that reduced expression of *TMPRSS2* has been linked with poor RFS prognosis in liver hepatocellular carcinoma, ovarian cancer, pheochromocytoma, stomach adenocarcinoma, paraganglioma, testicular germ cell tumour and uterine corpus endometrial carcinoma (Figure S3 and Table 2).

**TABLE 2:** Confirmation of the relationship between *TMPRSS2* expression and prognosis in various cancer patients (Kaplan–Meier plotter database)

	*n*	OS			PFS		
		*p*	HR	95% CI	*p*	HR	95% CI
Bladder carcinoma	405	0.0297	0.72	0.54–0.97	0.0895	0.55	0.27–1.11
Breast cancer	1090	0.0047	1.59	1.15–2.19	0.1543	0.73	0.48–1.13
Cervical squamous cell carcinoma	304	0.4036	0.82	0.51–1.32	0.155	1.75	0.80–3.81
Oesophageal adenocarcinoma	80	0.3012	0.7	0.36–1.37	0.0826	0.21	0.03–1.48
Oesophageal squamous cell carcinoma	81	0.2081	0.6	0.27–1.34	0.1914	0.44	0.13–1.55
Head–neck squamous cell carcinoma	500	0.0098	0.67	0.49–0.91	0.263	0.65	0.31–1.38
Kidney renal clear cell carcinoma	530	0.2005	1.23	0.90–1.67	0.0902	0.42	0.15–1.18
Kidney renal papillary cell carcinoma	288	0.0188	0.49	0.27–0.90	0.1444	1.75	0.82–3.74
Liver hepatocellular carcinoma	371	0.0321	0.67	0.46–0.97	0.0144	0.65	0.46–0.92
Lung adenocarcinoma	513	0.0002	0.58	0.44–0.78	0.2405	0.78	0.51–1.18
Lung squamous cell carcinoma	501	0.2355	1.18	0.9–1.55	0.4403	0.82	0.50–1.36
Ovarian cancer	374	0.0118	1.48	1.09–2.02	0.022	0.65	0.44–0.94
Pancreatic ductal adenocarcinoma	177	0.1197	1.52	0.89–2.59	0.1001	2.28	0.83–6.25
Pheochromocytoma and paraganglioma	178	0.28	2.56	0.44–14.88	0.0503	0	0–Inf
Rectum adenocarcinoma	165	0.0433	2.2	1–4.84	0.4488	0.54	0.1–2.75
Sarcoma	259	0.0051	1.8	1.19–2.73	0.3045	1.32	0.78–2.23
Stomach adenocarcinoma	375	0.0218	0.66	0.47–0.94	0.0422	0.51	0.27–0.99
Testicular germ cell tumour	134	0.168	4.33	0.45–42.02	0.0167	3.91	1.17–13.02
Thymoma	119	0.0246	0.18	0.03–0.93	–	–	–
Thyroid carcinoma	502	0.155	2.42	0.69–8.51	0.1234	0.55	0.25–1.19
Uterine corpus endometrial carcinoma	543	0.0002	0.46	0.31–0.70	0.0503	0.59	0.35–1.01

Abbreviations: 95% CI, confidence interval; HR, hazard rate; OS, overall survival; PFS, progression‐free survival.

### 3.6 The expression of *TMPRSS2* was associated with the immune infiltration in many carcinomas

**FIGURE 8:**
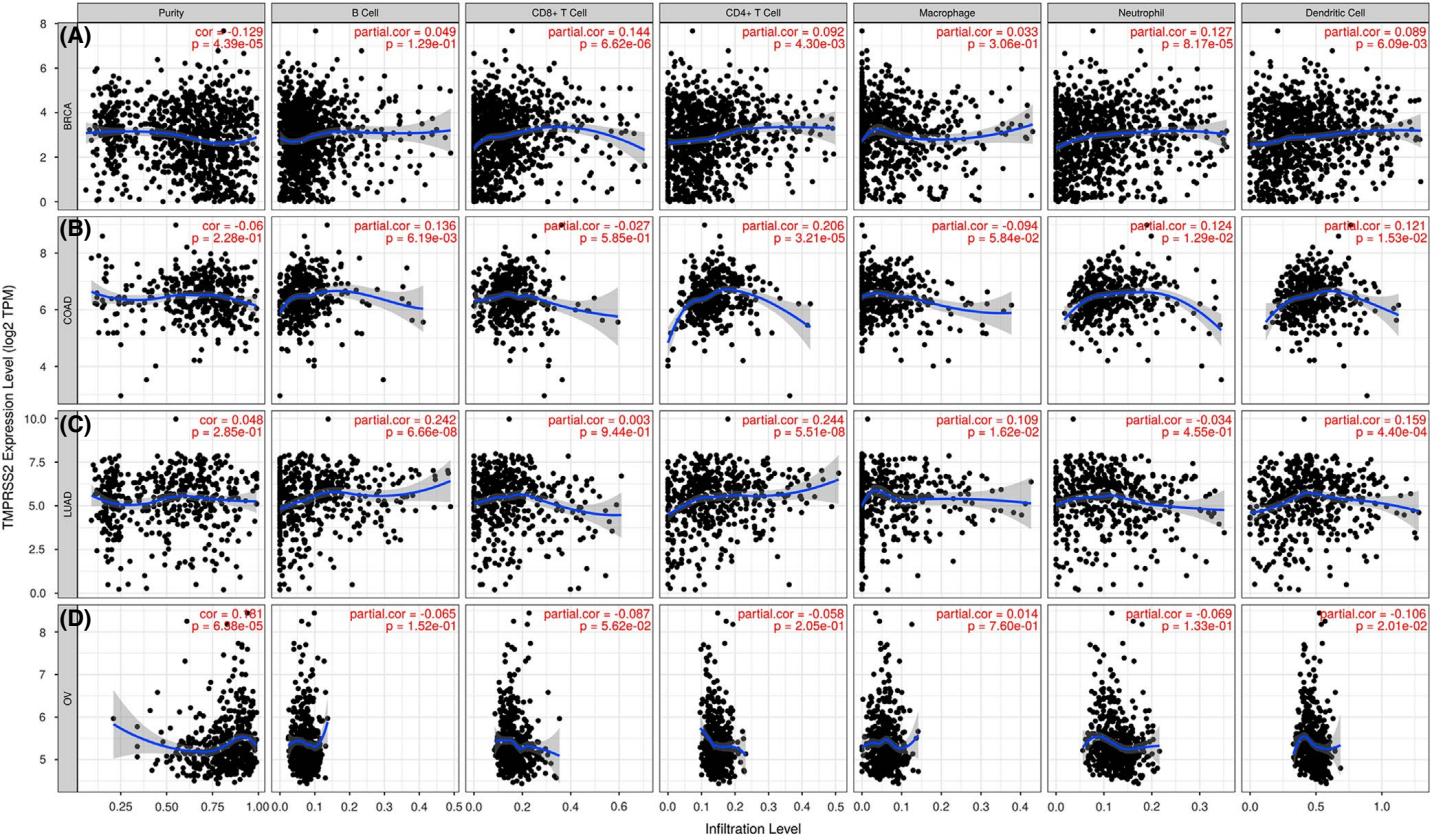
Correlation between *TMPRSS2* expression and immune infiltration in four common types of cancers, that is, lung, breast, ovarian and colorectal by TIMER database. The expression level of *TMPRSS2* has a considerable association with CD8+ T cell, tumour purity, dendritic cells and neutrophils in BRCA (A), B cell, CD4+ T cell, neutrophil and dendritic cell in COAD (B), CD4+ T cell, B cell, dendritic cells and macrophages in LUAD (C) and tumour purity and dendritic cell in OV (D). The *x*‐axis has been categorized based on immune infiltration and *y*‐axis indicates the expression level of *TMPRSS2* mRNA

Due to the abnormal expression and prognostic value of *TMPRSS2* in the lung, ovarian, breast and colorectal cancer, the underlined particular types of cancer were chosen to further identify that whether the expression level of *TMPRSS2* was associated with the level of immune infiltration via TIMER databases. The obtained results indicated that the expression level of *TMPRSS2* has a considerable association with tumour purity, neutrophils, CD8+ T cell and dendritic cell in BRCA; CD4+ T cell, B cell, DCs and neutrophils in COAD; B cell, macrophages, CD4+ T cell and dendritic cell in LUAD; and tumour purity and dendritic cell in OV respectively (Figure 8).

### 3.7 The association of differentially expressed genes with the expression of *TMPRSS2* in many cancers

**FIGURE 9:**
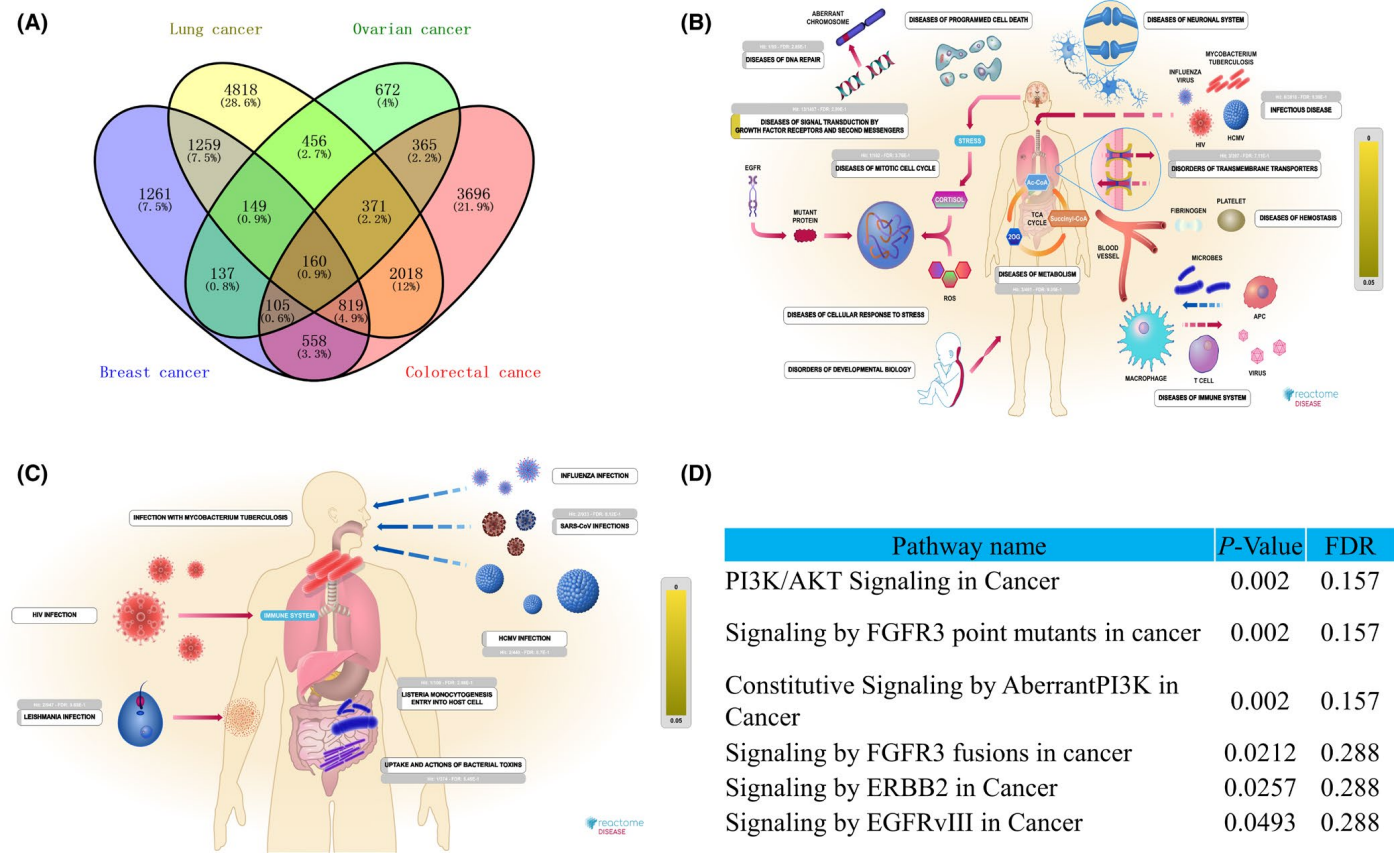
Analysis of positively correlated genes of *TMPRSS2* and their predicted cascade analysis by R2 and Reactome tools. (A) Venn diagram of 160 genes co‐expressed differentially and positively correlated to *TMPRSS2*, indicating coincident genes in lung, breast, ovarian and colorectal cancers. (B) 160 co‐expressed differential genes are effectively associated with the incidence and progression of human diseases. (C) 160 co‐expressed differential genes participated in respiratory infections such as influenza, SARS‐Cov, tuberculosis and HCMV. (D) Pathway analysis of *TMPRSS2* by Reactome and following a classification based on their cascades

We further explored the potential signalling mechanism and pathway linked with *TMPRSS2* in four common types of cancers (breast, lung, colorectal and ovarian) through the R2 platform and Reactome tool. The results revealed that 160 differently expressed genes (DEGs) were found in four selected cancers derived from the Venn diagram, as depicted in Figure 9A. The Reactome diseases analysis revealed that these 160 co‐expressed differential genes are considerably associated with the occurrence as well as the development of diseases including cell cycle monitoring, cell death, DNA repair, immune response, stress response and infection (Figure 9B). In terms of infectious diseases, their participation in respiratory infections, such as influenza, SARS‐Cov, tuberculosis and HCMV was particularly gaining attention (Figure 9C). In addition, the Reactome pathway analysis indicated that particularly associated genes were classified in FGFR3 point mutants, constitutive signalling by aberrant *PI3K*, *PI3K*/*AKT*, *NOTCH1 PEST* domain mutants, *NOTCH1*, *FGFR3* fusions, *NOTCH1 HD* + *PEST* domain mutants and *ERBB2* in cancer, as depicted in Figure 9D.

## 4 DISCUSSION

As the number of confirmed and death cases caused by COVID‐19 far exceeds any viral infection in the past, it has become a global pandemic and public health emergency of international concern.^35^ Epidemiological studies have shown that the population is generally susceptible to COVID‐19, but people with a low immune function, such as patients with cancer, diabetes, hypertension, autoimmune diseases, chronic obstructive pulmonary disease, organ transplants receiving anti‐immune therapy, the elderly and pregnant women are more susceptible to infection.^6^, ^36^ The death rate of cancer patients infected with COVID‐19 is as high as 5.6%.^7^, ^12^, ^14^, ^36^ Currently efforts have been directed towards studying the early screening, isolation measures and treatment of COVID‐19, but the relationship and mechanism between COVID‐19 and cancer remain unclear.

Cumulative studies have reported that the process of SARS‐CoV‐2 entering the host alveolar epithelial cells is closely related to angiotensin‐converting enzyme 2 (*ACE2*) and *TMPRSS2*.^18^, ^37^ Among them, *TMPRSS2* is a key molecular target that enhances the invading capacity of SARS‐CoV‐2, has been earmarked as candidate drug targets for interventions against the viral pathologies.^18^ Besides its role in COVID‐19, *TMPRSS2* considerably contributes to several physiological and pathological processes, included in tumour biology.^19^, ^20^ It was worth noting that *TMPRSS2* is not only closely associated with the occurrence and progression of prostate cancer, but also can be used as an attractive therapeutic target.^20^ It has been reported that *TMPRSS2* has a key role in many kinds of cancers in vitro and in vivo including breast,^38^ colorectal,^39^ head and neck,^22^ ovarian,^40^ stomach and lung cancer.^41^ However, the expression and prognostic value of *TMPRSS2* in many cancers have not been clearly defined. In this study, we evaluated the expression of *TMPRSS2* that might be responsible for the COVID‐19 condition in patients with different types of cancers. Analysis of datasets indicated that the expression level of *TMPRSS2* was considerably reduced in many tumour tissues, relative to their normal counterparts, and the level of *TMPRSS2* expression was considerably associated with the prognosis of the brain, blood, colorectal, breast, ovarian, lung and soft tissue cancer. Although a large number of studies on the *TMPRSS2*, ERG fusion gene are considered to be new biomarkers, therapeutic targets, diagnostic and prognostic indicators for prostate cancer, and two recent studies have used public databases to explore the expression pattern, prognostic value and immune infiltration of *TMPRSS2* in prostate cancer, but these studies are limited to prostate cancer and cannot fully reflect all tumours.^42^, ^43^ Unfortunately, neither of the PrognoScan and Kaplan–Meier plotter databases used in our research are involved prostate cancer data.^44^ In the future, we will further explore the difference between the *TMPRSS2*: ERG fusion gene and the *TMPRSS2* gene through basic and clinical research, as well as the prognostic role in prostate cancer. Based on the above conclusions, we chose four common cancer types to further investigate the expression, promoter methylation, prognosis, mutation, protein–protein interaction analysis and immune infiltration levels of *TMPRSS2*.

In the case of breast cancer, our analysis showed that *TMPRSS2* had significantly low expression and low mutation frequency in tumour tissues, but its promoter methylation level was significantly increased. In addition, multiple datasets showed that patients with up‐regulated *TMPRSS2* expression had significantly bad OS, RFS and DMFS in patients with most tumour types. The underlined results are in favour of the earlier results where Chi et al.^38^ revealed that dexmedetomidine may contribute to the regulation of the migration of breast cancer cells by up‐regulation of *TMPRSS2*. Meanwhile, a recent study pointed out that lower expression of *TMPRSS2* is a prognostic marker for OS and DFS in breast cancer.^45^ In colorectal cancer, the results of previous studies are similar to those of our study. For example, the study of Liu et al.^39^ found an elevated expression of *TMPRSS2* in colorectal tumour and normal tissue samples, relative to other tumours, such as lung, oesophagus, stomach and liver; Bao et al.^46^ revealed that the *TMPRSS2* expression was considerably elevated in normal tissues than colorectal cancers using across several existing databases. As for lung cancer, one single nucleus and single‐cell RNA sequencing study showed a strong expression level of *TMPRSS2* in lung tissue and cells derived from subsegmental bronchial branches, and smoking may be susceptible to COVID‐19 by affecting the expression of *TMPRSS2*.^47^, ^48^ In addition, both our study and previous studies believed that *TMPRSS2* as a cancer suppressor gene was significantly down‐regulated in two types of tumour tissues of LUAD and LUSC, the expression of *TMPRSS2* have an impact on the prognosis of lung cancer.^49^ As for ovarian cancer, Huang et al. observed the oncogenic gene fusion *TMPRSS2*: ERG has not been present in ovarian cancer. Therefore, ERG cannot be used as a potential diagnostic or prognostic indicator for ovarian cancer, unlike prostate cancer.^40^ Unfortunately, there is little known on the prognostic value and molecular mechanism of *TMPRSS2* in ovarian cancer. In our analysis, we found that higher OS and DFS were indicated in the low *TMPRSS2* expression group of ovarian cancer relative to the elevated expression group, based on DUKE‐OC, GSE9891 and GSE26712 dataset, but the specific mechanism needs further verification and discussion in the future.

To better explore the internal connection of *TMPRSS2*, COVID‐19 and tumour, we exploited GeneMANIA and STRING webserver to predict the functional protein partners of *TMPRSS2*. A total of 27 genes or proteins were determined as gene and protein partners of *TMPRSS2*, which might contribute to regulating the progression of carcinoma mediated by *TMPRSS2* and its survival rate. Then, the mutations and CNAs were analysed in the 27 predicted protein‐related coding genes of *TMPRSS2* via the cBioPortal database, the results showed that these genes were altered in 38% (3225/10,953) queried cancer patients. Furthermore, the association between *TMPRSS2* expression and the level of immune infiltration was also determined; our results indicated that an elevated level of *TMPRSS2* was linked with infiltration of immune cells in BRCA, COAD, LUAD and OV. The underlined results showed similarity with the results of Luo et al.^42^ which revealed that elevated expression levels of *TMPRSS2* mRNA were linked with an elevated immune infiltration in PRAD. Next, we evaluated the genes associated with *TMPRSS2* in several types of cancer, that is, lung, breast, colorectal and ovarian cancer by using the R2 platform. Our analysis has shown that 160 co‐expressed differential and positively correlated genes were found in the above four common cancers. According to the pathway analysis, these genes are significantly enriched in associated cascades including respiratory system infection and tumour progression, especially the multiple viral infection pathways and *PI3K*/*AKT* signalling pathways are worthy of attention.

## 5 CONCLUSIONS

In our study, the expression, promoter methylation, genetic variation, protein partners, associated genes, immune infiltration and prognostic value of *TMPRSS2* were systematically analysed in many kinds of cancer in humans. These results indicated that *TMPRSS2* expression was decreased in many tumour tissues distinctively and was significantly related to the clinical outcomes in the brain, blood, colorectal, breast, lung, soft tissue and ovarian cancer. The multi‐omics analysis also revealed the significance of *TMPRSS2* expression and possible cascades associated with *TMPRSS2* in several kinds of cancers (human) and progression of COVID‐19. Hence, the underlined results may help to explore *TMPRSS2* as an effective biomarker and therapeutic target for many kinds of cancer in humans, and also provide directions for the prevention of COVID‐19 pandemic for particular tumour patients.

## CONFLICT OF INTERESTS

The authors declare no conflict of interest.

## AUTHOR CONTRIBUTIONS

**Li Liu:** Conceptualization (lead); Formal analysis (supporting); Investigation (supporting); Project administration (supporting); Resources (supporting); Software (supporting); Supervision (supporting). **Ju‐Fang Qin:** Formal analysis (supporting); Methodology (supporting); Software (supporting). **Man‐Zhen Zuo:** Conceptualization (supporting); Data curation (supporting); Investigation (supporting); Software (supporting); Supervision (supporting); Writing – review & editing (supporting). **Quan Zhou:** Conceptualization (lead); Data curation (lead); Methodology (lead); Resources (lead); Supervision (lead); Writing – original draft (lead); Writing – review & editing (lead).

## Supporting information

Figure S1

Figure S2

Figure S3

## Data Availability

All data generated or analysed during this study are included in this article.

## References

[1] Sung H , Ferlay J , Siegel RL , et al. Global Cancer Statistics 2020: GLOBOCAN estimates of incidence and mortality worldwide for 36 cancers in 185 countries. CA Cancer J Clin. 2021;71(3):209‐249. doi:10.3322/caac.21660 33538338

[2] Bray F , Jemal A , Grey N , Ferlay J , Forman D . Global cancer transitions according to the Human Development Index (2008–2030): a population‐based study. Lancet Oncol. 2012;13:790‐801. doi:10.1016/S1470-2045(12)70211-5 22658655

[3] Rebbeck TR , Burns‐White K , Chan AT , et al. Precision prevention and early detection of cancer: fundamental principles. Cancer Discov. 2018;8:803‐811. doi:10.1158/2159-8290.CD-17-1415 29907587

[4] Center JHUCR Global Map. 1 November 2021. Available online: https://coronavirus.jhu.edu/. Accessed November 1 2021.

[5] Yuce M , Filiztekin E , Ozkaya KG . COVID‐19 diagnosis – a review of current methods. Biosens Bioelectron. 2021;172:112752. doi:10.1016/j.bios.2020.112752 33126180 PMC7584564

[6] Brix TH , Hegedus L , Hallas J , Lund LC . Risk and course of SARS‐CoV‐2 infection in patients treated for hypothyroidism and hyperthyroidism. Lancet Diabetes Endocrinol. 2021;9(4):197‐199. doi:10.1016/S2213-8587(21)00028-0 33617779 PMC7906640

[7] Desai A , Sachdeva S , Parekh T , Desai R . COVID‐19 and cancer: lessons from a pooled meta‐analysis. JCO Glob Oncol. 2020;6:557‐559. doi:10.1200/GO.20.00097 32250659 PMC7193801

[8] Fang X , Li S , Yu H , et al. Epidemiological, comorbidity factors with severity and prognosis of COVID‐19: a systematic review and meta‐analysis. Aging (Albany NY). 2020;12:12493‐12503. doi:10.18632/aging.103579 32658868 PMC7377860

[9] Guan WJ , Liang WH , He JX , Zhong NS . Cardiovascular comorbidity and its impact on patients with COVID‐19. Eur Respir J. 2020;55:2001227. doi:10.1183/13993003.01227-2020 32341104 PMC7236831

[10] Liang W , Guan W , Chen R , et al. Cancer patients in SARS‐CoV‐2 infection: a nationwide analysis in China. Lancet Oncol. 2020;21:335‐337. doi:10.1016/S1470-2045(20)30096-6 32066541 PMC7159000

[11] Zheng RS , Sun KX , Zhang SW , et al. Report of cancer epidemiology in China, 2015. Zhonghua Zhong Liu Za Zhi. 2019;41:19‐28. doi:10.3760/cma.j.issn.0253-3766.2019.01.005 30678413

[12] Moujaess E , Kourie HR , Ghosn M . Cancer patients and research during COVID‐19 pandemic: a systematic review of current evidence. Crit Rev Oncol Hematol. 2020;150:102972. doi:10.1016/j.critrevonc.2020.102972 32344317 PMC7174983

[13] Saini KS , Tagliamento M , Lambertini M , et al. Mortality in patients with cancer and coronavirus disease 2019: a systematic review and pooled analysis of 52 studies. Eur J Cancer. 1990;2020(139):43‐50. doi:10.1016/j.ejca.2020.08.011 PMC7467090 32971510

[14] Xia Y , Jin R , Zhao J , Li W , Shen H . Risk of COVID‐19 for patients with cancer. Lancet Oncol. 2020;21:e180. doi:10.1016/S1470-2045(20)30150-9 32142622 PMC7130057

[15] Chen YW , Lee MS , Lucht A , et al. *TMPRSS2*, a serine protease expressed in the prostate on the apical surface of luminal epithelial cells and released into semen in prostasomes, is misregulated in prostate cancer cells. Am J Pathol. 2010;176:2986‐2996. doi:10.2353/ajpath.2010.090665 20382709 PMC2877858

[16] Shen LW , Mao HJ , Wu YL , Tanaka Y , Zhang W . *TMPRSS2*: a potential target for treatment of influenza virus and coronavirus infections. Biochimie. 2017;142:1‐10. doi:10.1016/j.biochi.2017.07.016 28778717 PMC7116903

[17] Stopsack KH , Mucci LA , Antonarakis ES , Nelson PS , Kantoff PW . *TMPRSS2* and COVID‐19: serendipity or opportunity for intervention? Cancer Discov. 2020;10:779‐782. doi:10.1158/2159-8290.CD-20-0451 32276929 PMC7437472

[18] McKee DL , Sternberg A , Stange U , Laufer S , Naujokat C . Candidate drugs against SARS‐CoV‐2 and COVID‐19. Pharmacol Res. 2020;157:104859. doi:10.1016/j.phrs.2020.104859 32360480 PMC7189851

[19] Bahmad HF , Abou‐Kheir W . Crosstalk between COVID‐19 and prostate cancer. Prostate Cancer Prostatic Dis. 2020;23:561‐563. doi:10.1038/s41391-020-0262-y 32709978 PMC7378980

[20] Bhowmick NA , Oft J , Dorff T , et al. COVID‐19 and androgen‐targeted therapy for prostate cancer patients. Endocr Relat Cancer. 2020;27:R281‐R292. doi:10.1530/ERC-20-0165 32508311 PMC7546583

[21] Bestle D , Heindl MR , Limburg H , et al. *TMPRSS2* and furin are both essential for proteolytic activation of SARS‐CoV‐2 in human airway cells. Life Sci Alliance. 2020;3:e202000786. doi:10.26508/lsa.202000786 32703818 PMC7383062

[22] Sacconi A , Donzelli S , Pulito C , et al. *TMPRSS2*, a SARS‐CoV‐2 internalization protease is downregulated in head and neck cancer patients. J Exp Clin Cancer Res. 2020;39:200. doi:10.1186/s13046-020-01708-6 32967703 PMC7510014

[23] Lucas JM , Heinlein C , Kim T , et al. The androgen‐regulated protease *TMPRSS2* activates a proteolytic cascade involving components of the tumor microenvironment and promotes prostate cancer metastasis. Cancer Discov. 2014;4:1310‐1325. doi:10.1158/2159-8290.CD-13-1010 25122198 PMC4409786

[24] Rhodes DR , Kalyana‐Sundaram S , Mahavisno V , et al. Oncomine 3.0: genes, pathways, and networks in a collection of 18,000 cancer gene expression profiles. Neoplasia. 2007;9:166‐180. doi:10.1593/neo.07112 17356713 PMC1813932

[25] Tang Z , Li C , Kang B , Gao G , Li C , Zhang Z . GEPIA: a web server for cancer and normal gene expression profiling and interactive analyses. Nucleic Acids Res. 2017;45:W98‐W102. doi:10.1093/nar/gkx247 28407145 PMC5570223

[26] Chandrashekar DS , Bashel B , Balasubramanya SAH , et al. UALCAN: a portal for facilitating tumor subgroup gene expression and survival analyses. Neoplasia. 2017;19:649‐658. doi:10.1016/j.neo.2017.05.002 28732212 PMC5516091

[27] Gao J , Aksoy BA , Dogrusoz U , et al. Integrative analysis of complex cancer genomics and clinical profiles using the cBioPortal. Sci Signal. 2013;6:pl1. doi:10.1126/scisignal.2004088 23550210 PMC4160307

[28] Nagy A , Gyorffy B . muTarget: a platform linking gene expression changes and mutation status in solid tumors. Int J Cancer. 2021;148:502‐511. doi:10.1002/ijc.33283 32875562

[29] Li T , Fan J , Wang B , et al. TIMER: a web server for comprehensive analysis of tumor‐infiltrating immune cells. Can Res. 2017;77:e108‐e110. doi:10.1158/0008-5472.CAN-17-0307 PMC6042652 29092952

[30] Mizuno H , Kitada K , Nakai K , Sarai A . PrognoScan: a new database for meta‐analysis of the prognostic value of genes. BMC Med Genomics. 2009;2:18. doi:10.1186/1755-8794-2-18 19393097 PMC2689870

[31] Gyorffy B , Lanczky A , Eklund AC , et al. An online survival analysis tool to rapidly assess the effect of 22,277 genes on breast cancer prognosis using microarray data of 1,809 patients. Breast Cancer Res Treat. 2010;123(3):725‐731. doi:10.1007/s10549-009-0674-9 20020197

[32] Warde‐Farley D , Donaldson SL , Comes O , et al. The GeneMANIA prediction server: biological network integration for gene prioritization and predicting gene function. Nucleic Acids Res. 2010;38:W214‐W220. doi:10.1093/nar/gkq537 20576703 PMC2896186

[33] Szklarczyk D , Franceschini A , Wyder S , et al. STRING v10: protein‐protein interaction networks, integrated over the tree of life. Nucleic Acids Res. 2015;43:D447‐D452. doi:10.1093/nar/gku1003 25352553 PMC4383874

[34] Vastrik I , D'Eustachio P , Schmidt E , et al. Reactome: a knowledge base of biologic pathways and processes. Genome Biol. 2007;8:R39. doi:10.1186/gb-2007-8-3-r39 17367534 PMC1868929

[35] Giannis D , Ziogas IA , Gianni P . Coagulation disorders in coronavirus infected patients: COVID‐19, SARS‐CoV‐1, MERS‐CoV and lessons from the past. J Clin Virol. 2020;127:104362. doi:10.1016/j.jcv.2020.104362 32305883 PMC7195278

[36] Guan WJ , Liang WH , Zhao Y , et al. Comorbidity and its impact on 1590 patients with COVID‐19 in China: a nationwide analysis. Eur Respir J. 2020;55:2000547. doi:10.1183/13993003.00547-2020 32217650 PMC7098485

[37] Dong M , Zhang J , Ma X , et al. ACE2, *TMPRSS2* distribution and extrapulmonary organ injury in patients with COVID‐19. Biomed Pharmacother. 2020;131:110678. doi:10.1016/j.biopha.2020.110678 32861070 PMC7444942

[38] Chi M , Shi X , Huo X , Wu X , Zhang P , Wang G . Dexmedetomidine promotes breast cancer cell migration through Rab11‐mediated secretion of exosomal *TMPRSS2* . Ann Transl Med. 2020;8:531. doi:10.21037/atm.2020.04.28 32411754 PMC7214880

[39] Liu C , Wang K , Zhang M , et al. High expression of ACE2 and *TMPRSS2* and clinical characteristics of COVID‐19 in colorectal cancer patients. NPJ Precis Oncol. 2021;5:1. doi:10.1038/s41698-020-00139-y 33479506 PMC7820314

[40] Huang L , Schauer IG , Zhang J , et al. The oncogenic gene fusion *TMPRSS2*: ERG is not a diagnostic or prognostic marker for ovarian cancer. Int J Clin Exp Pathol. 2011;4:644‐650. 22076164 PMC3209604

[41] Hoang T , Nguyen TQ , Tran TTA . Genetic susceptibility of ACE2 and *TMPRSS2* in six common cancers and possible impacts on COVID‐19. Cancer Res Treat. 2020;53(3):650–656. doi:10.4143/crt.2020.950 33421977 PMC8291170

[42] Luo L , Zheng Y , Li M , et al. *TMPRSS2* correlated with immune infiltration serves as a prognostic biomarker in prostatic adenocarcinoma: implication for the COVID‐2019. Front Genet. 2020;11:575770. doi:10.3389/fgene.2020.575770 33193689 PMC7556306

[43] Cheng J , Zhou J , Fu S , et al. Prostate adenocarcinoma and COVID‐19: the possible impacts of *TMPRSS2* expressions in susceptibility to SARS‐CoV‐2. J Cell Mol Med. 2021;25:4157‐4165. doi:10.1111/jcmm.16385 33609069 PMC8013364

[44] Garcia‐Perdomo HA , Chaves MJ , Osorio JC , Sanchez A . Association between *TMPRSS2*:ERG fusion gene and the prostate cancer: systematic review and meta‐analysis. Cent European J Urol. 2018;71:410‐419. doi:10.5173/ceju.2018.1752 PMC6338815 30680235

[45] Parmar HS , Nayak A , Gavel PK , Jha H , Bhagwat S , Sharma R . Cross talk between COVID‐19 and breast cancer. Curr Cancer Drug Targets. 2021;21(7):575‐600. doi:10.2174/1568009621666210216102236 33593260

[46] Bao R , Hernandez K , Huang L , Luke JJ . ACE2 and *TMPRSS2* expression by clinical, HLA, immune, and microbial correlates across 34 human cancers and matched normal tissues: implications for SARS‐CoV‐2 COVID‐19. J Immunother Cancer. 2020;8:e001020. doi:10.1136/jitc-2020-001020 32675312 PMC7372174

[47] Lukassen S , Chua RL , Trefzer T , et al. SARS‐CoV‐2 receptor ACE2 and *TMPRSS2* are primarily expressed in bronchial transient secretory cells. EMBO J. 2020;39:e105114. doi:10.15252/embj.20105114 32246845 PMC7232010

[48] Yin J , Kasper B , Petersen F , Yu X . Association of cigarette smoking, COPD, and lung cancer with expression of SARS‐CoV‐2 entry genes in human airway epithelial cells. Front Med (Lausanne). 2020;7:619453. doi:10.3389/fmed.2020.619453 33425965 PMC7793919

[49] Wu X , Wang L , Feng F , Tian S . Weighted gene expression profiles identify diagnostic and prognostic genes for lung adenocarcinoma and squamous cell carcinoma. J Int Med Res. 2020;48:300060519893837. doi:10.1177/0300060519893837 31854219 PMC7607763

