# Supplementary figures and images for: Multi‐omics of the expression and clinical outcomes of TMPRSS2 in human various cancers: A potential therapeutic target for COVID‐19
Source: J Cell Mol Med. 2021 Dec 24;26(3):709–24. doi: 10.1111/jcmm.17090 (PMC8817140; doi:10.1111/jcmm.17090)

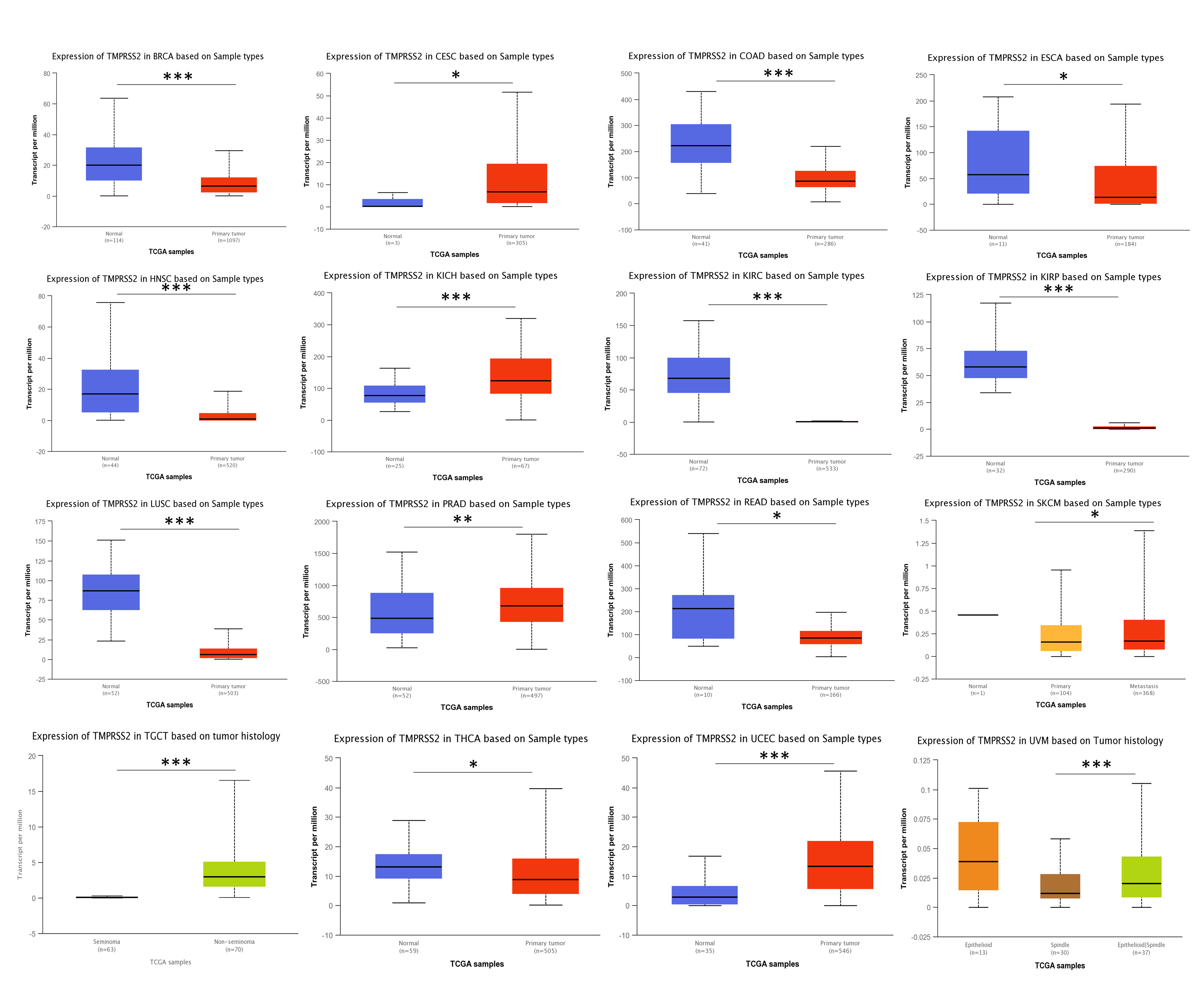

Supplement: Supplementary file 1 — Figure S1 [file JCMM-26-709-s003.tif]

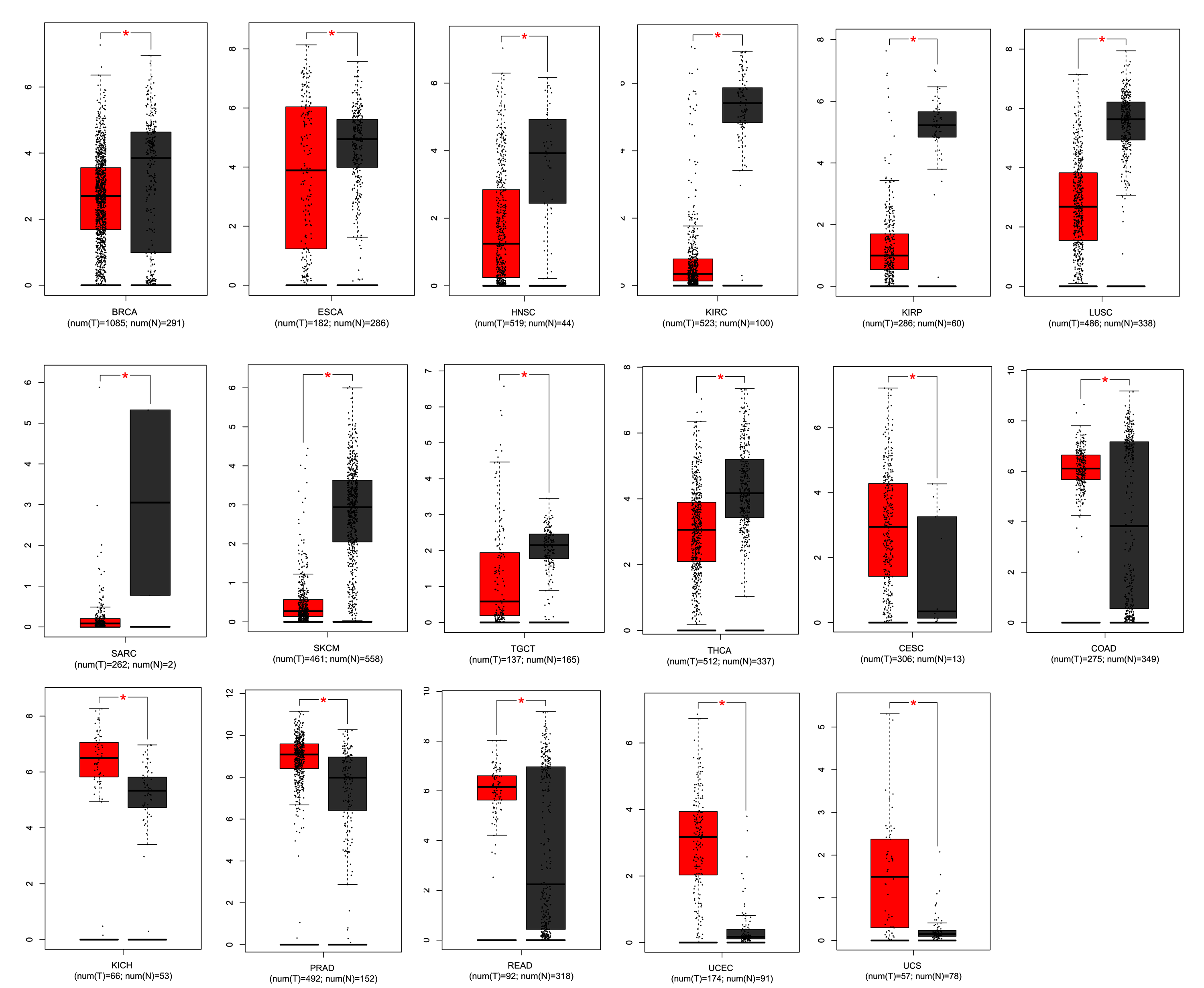

Supplement: Supplementary file 2 — Figure S2 [file JCMM-26-709-s001.tiff]

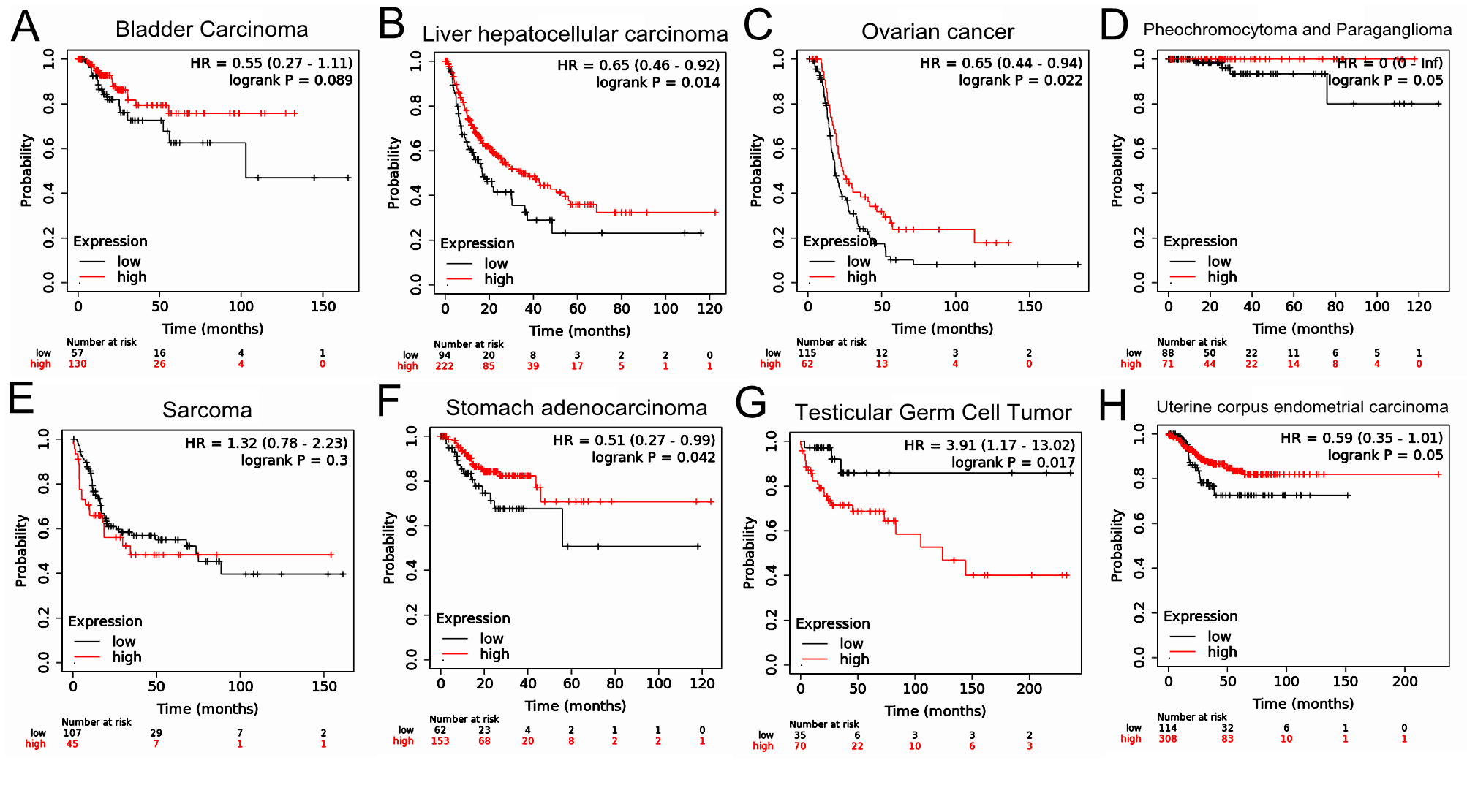

Supplement: Supplementary file 3 — Figure S3 [file JCMM-26-709-s002.tiff]
